# Employment and Mental Health of the Chinese Elderly: Evidence from CHARLS 2018

**DOI:** 10.3390/ijerph20042791

**Published:** 2023-02-04

**Authors:** Yanrong Cheng, Jian Lan, Qinying Ci

**Affiliations:** 1 School of Sociology, Wuhan University, Wuhan 430072, China; 2 College of State Governance, Southwest University, Chongqing 400715, China

**Keywords:** employment, mental health, Chinese older people, individual annual income, financial support provided to children, support received from children

## Abstract

Improving the mental health of the elderly has become an important strategic goal of healthy aging, among which employment is regarded as an essential factor for the mental health of the elderly. This study adopted ordinary least squares, ordered logit, propensity score matching (PSM), and KHB mediation analysis to examine the influence and mechanism of employment on mental health in older Chinese adults using data from the China Health and Retirement Longitudinal Survey of 2018. The study found that employment positively impacts older adults’ mental health in China. This promotive effect of employment was significant for more senior people aged up to 80 years old with lower educational backgrounds and rural household registration. In addition, individual annual income, the financial support provided to children, and support received from children significantly mediate the realization of employment improving older people’s mental health. Our findings are expected to provide valuable insight into delayed retirement and active aging in China. Therefore, the government must play the role of support and advocacy to promote employment and safeguard the well-being of older adults.

## 1. Introduction

Population aging is becoming a universal trend, an inevitable consequence of economic and social progress, resulting from the combined effect of low fertility and longer life expectancy. Notably, China, a developing country with a larger population and stricter family planning policies, is facing a more difficult aging situation. In 2000, an aging society with more than 7% of the population aged 65 and above came to China, and it has already become a profoundly aging society with more than 14% of the elderly population in 2021, which means that China’s aging population is rapidly growing. Meanwhile, along with the accelerating rate of aging in China, the scale of the working-age population is gradually decreasing, the burden of pensions is increasing, the dependency ratio is rising, and a potential shortage of social pension insurance funds is becoming increasingly severe. Against such a background, encouraging the elderly to stay in the labor market and developing human resources for the elderly have become a necessity for the Chinese government through the policy of proactively responding to population aging and delaying retirement.

Increasingly, scholars have been concerned about the psychological well-being of elders, especially depression, which is becoming a common mood disorder among the elderly in China [1,2,3,4,5,6]. The China Health and Retirement Report indicated that about 33.1% of the Chinese population over age 60 was at high risk of depression [7]. The improvement of the mental health of the elderly has become an important strategic goal of healthy aging. The change in social roles and lifestyles due to retirement can easily lead to depression and other mental health problems among the elderly [8]. Therefore, employment is regarded as an essential factor affecting the mental health of older people [9].

The published studies have shown controversial results concerning the impact of employment on the mental health of older adults. Mainly, the health promotion and the health aggravation hypotheses are two perspectives. The health promotion hypothesis has held that employment plays a protective role against depressive symptoms among older adults [10,11,12,13,14]. These studies have shown that the mechanism is that work represents the achievement of personal status and the continuation of social roles, and withdrawal from the labor market or retirement is a symbol of losing identity and functions, which may deteriorate the mental health of the elderly [15]. Moreover, based on the social support theory, older people are likely to have lower levels of social support and fewer opportunities for social engagement after retirement, resulting in adverse effects on their mental health [16]. The continuity theory also holds that retirees are motivated to achieve role continuity to maintain or improve their satisfaction and well-being. Continuing to work is one of the ways to keep the retiree’s original life pattern [11,17,18,19]. Therefore, employment can reduce depressive symptoms and promote mental health among older adults compared with those who are not employed.

However, some of the existing literature has found that employment is negatively associated with elders’ mental health, and retirement helps improve their physical and psychological health [9,20,21,22]. Firstly, engagement takes up the leisure time of the elderly so that they cannot engage in exercise and volunteer service, which will harm their mental health [22,23]. Because of the influence of the traditional family culture, older people in China prefer peaceful old age. However, work symbolizes tiredness and pressure, and employment will lead to a decline in life satisfaction [24]. Lastly, it has been suggested that normal retirement does not adversely affect the health of older adults but rather that continued work can lead to a deterioration in mental health [25,26,27].

Although there are no consistent conclusions about older adults’ employment and mental health, the existing studies have provided relevant references. However, in general, the current studies had the following shortcomings: some reflections on the relationship between employment and depression are primarily from Western developed countries, but relevant studies in developing countries, such as China, are still scarce. Moreover, even if there are China-related studies, they focus more on urban samples [9,22,23,28], with little attention to rural areas. Furthermore, their studies cannot conduct an in-depth analysis of the mechanism and consider endogeneity problems resulting in self-selection bias. Thus, this study puts forward a topic to be discussed, that is, whether the employment of older Chinese people will help to improve their mental health. If there is a positive connection between the two, is there a difference in this connection among different groups? If so, then what is its mechanism?

With the above in mind, we proposed the hypothesis as follows:

**Hypothesis** **1.** *Employment will benefit the mental health of older adults*.

Some scholars have found that the impact of working after retirement on mental health was heterogeneous concerning individual characteristics [9,11,14,22]. Moreover, it is necessary to analyze the impact on different groups. Therefore, the following hypothesis can be proposed:

**Hypothesis** **2.** *The impact of employment on the mental health of older adults is heterogeneous in different groups*.

In a further step, we would like to investigate the influence mechanism between employment and the mental health of older adults. Unlike in Western countries, where the primary purpose of working for older people is to achieve self-realization, there has been a controversy over whether the aim of employment for older people in China is "self-need" or "family need." With urbanization and the growing influence of individualism in China, a large number of children are living far from home and working outside, and support from the elderly’s children is becoming less reliable. Thus, the elderly secure their pension by earning income through employment [1,28]. On the other hand, having a traditional Confucian culture emphasizes substantial family obligations and intergenerational solidarity, which remain central in Chinese family life [29].

Meanwhile, the structure of how filial commitments are understood and fulfilled has fundamentally changed. The intergenerational relationship has been replaced by a historical hierarchy to emphasize mutual care and reciprocal exchanges, reflecting an "intergenerational contract" that has been renegotiated and reinterpreted by both generations in support of a robust and reciprocated cycle of care [30]. As a result, most elders will rely on themselves to solve their financial problems while still remaining healthy. That is, they continue to participate in the workforce to ease the budgetary constraints of their children and reduce their financial stress. In turn, children provide feedback to their parents by providing care and emotional support, both material and non-material, to an extent. The above analysis suggests that, on the one hand, the employment of older adults earns income and obtains livelihood security, and on the other hand, the elderly, who have a stable long-term source of income and adequate self-pension security, provide some financial assistance to their children through intergenerational support. Harmonious intergenerational relationships, in turn, have a positive impact on the mental health of the elderly and reduce their tendency to depression. Thus, we hypothesized the following:

**Hypothesis** **3.** *Employment will affect the mental health of older adults by affecting individual annual income, the financial support provided to children, and support received from children in the future*.

## 2. Data and Methods

### 2.1. Data

The data used in this study were from the China Health and Retirement Longitudinal Survey (CHARLS) of 2018, a large-scale interdisciplinary survey project hosted by the National Development Research Institute of Peking University. It aimed to collect a high-quality, nationally representative sample of Chinese residents ages 45 and older to serve the needs of scientific research on the elderly. CHARLS adopted multi-stage stratified PPS sampling. We obtained the data from the official website http://charls.pku.edu.cn (accessed on 6 August 2022), which was available to users worldwide.

Regarding the Law of the People’s Republic of China on the Protection of the Rights and Interests of the Elderly, this paper selected samples aged 60 and above and retained 4316 pieces after screening variables and cleaning data.

### 2.2. Variables

#### 2.2.1. Dependent Variable

The dependent variable was mental health, as measured by depression. The CHARLS 2018 questionnaire included the Center for Epidemiologic Studies Depression Scale (CES-D), a commonly used international scale for measuring depression according to the existing research [9,31,32], and this study used the CES-D to measure mental health. Based on the CES-D scale in the 2018 CHARLS data, negative emotions and behaviors were classified based on answers to the following questions: “I was bothered by things that don’t usually bother me,” “I had trouble keeping my mind on what I was doing,” “I felt depressed,” “I felt everything I did was an effort,” “I felt fearful,” “My sleep was restless.”, “I felt lonely,” and “I could not get ’going’.” The answers correspond to four options: rarely or none of the time (<1 day), some or a little of the time (1–2 days), occasionally or a moderate amount of the time (3–4 days), and most or all of the time (5–7 days), which were assigned values of 1, 2, 3, and 4, respectively. “I felt hopeful about the future” and “I was happy” reflected positive emotions, which were assigned in reverse. The total score of the CES-D range between 10 and 40. The higher the score, the worse the mental health, and vice versa.

#### 2.2.2. Independent Variable

The core independent variable was “employment," whether the older people continued to work or found new work. Work was defined as activities for earning a livelihood in the CHARLS 2018, including non-agricultural employment, non-agricultural self-employment, unpaid help for the family business, and agricultural employment. According to the existing research [9,28], if the respondents were in nonfarm or agrarian employment, the variable was set as 1, whereas if the respondents quit the labor market, it was established as 0.

#### 2.2.3. Covariates

Considering that other factors may confound the estimates of the effect of employment on the mental health of the elderly, this study selected the individual, family, and social characteristics of the respondents as control variables. The variables for individual characteristics include age, gender, years of education, marital status, and health status. The variables for family characteristics include annual household income and the total number of children. The variables for social characteristics include receiving pension status and household registration (Hukou).

#### 2.2.4. Mediating Variables

If employment significantly impacts the mental health of older adults, then we need to investigate the mechanism. Work may help older adults increase their income and provide children with financial support. Second, according to activity theory, work may increase opportunities for interpersonal communication and help to maintain positive emotional, caregiving, and financial support received from children, thereby improving the health of older adults. Based on previous related research [9,28,29], this study examined the mechanism of employment on mental health through the three aspects of individual annual income, the financial support provided to children, and support received from children. Individual annual income was measured by income self-evaluation, the financial support provided to children was measured by self-rated financial support provided to children, and support received from children was measured by self-rated emotional support, financial support, and caregiving received from children (see Table 1).

### 2.3. Models

#### 2.3.1. Basic Model

To empirically explore the effect of employment on the mental health of older adults, and the dependent variable was continuous, the model was set as follows:

(1) $$Depression_{i}=\alpha_{0}+\alpha_{1}Employment_{i}+\alpha_{2}X_{i}+\varepsilon_{i},$$

where the dependent variable $\;Depression_{i}$ was the mental health of individual i in the survey, $Employment_{i}\;$ indicated employment for individual i, $X_{i}$ was a series of control variables, and $\varepsilon_{i}$ was the random error term. First, depression was considered a continuous variable in this study, so OLS was adopted in the first step of the investigation. In addition, referring to Xie et al. [9], who regarded the explained variables as ordinal variables, we used the ordered logit regression model to augment the robustness of estimation results.

#### 2.3.2. Propensity Score Matching

The employment of older people was a self-selection behavior, as work was not randomly assigned to the older adults, resulting endogeneity problem. If it was not effectively handled, the impact of employment on mental health in the regression results might not have internal validity and may bias our main results. PSM could overcome the endogenous threat caused by self-selection and check the robustness of the main results. The participants ‘average treatment effect (ATT) was obtained through the following model:

(2) $$ATT={\;E[Y}_{1\;i}\;|\;D_{i}=1,\;p\left(X_{i}\right)]-{E[Y}_{0\;i}\;|\;D_{i}=0,\;p\left(X_{i}\right)].$$

Here, where *D* is the dummy variable (1 for the treated group, 0 for the control group), $Y_{1\;i}\;$ is the mental health of the treated group; $Y_{0\;i}$ is the depression score of the control group, and $X_{i}\;$ represents the covariates. $D_{i}=1$ indicated that the elderly were employed while $D_{i}=0$ meant that the older people were not employed. The PSM method provided an estimate with less selection bias between a treatment group and a control group. The goal is achieved by matching participants in the treatment group to similar participants drawn from a control sample of the demographic. Therefore, any observed differences between the treatment and control groups are most likely attributable to treatment effects and not to other confounding factors.

#### 2.3.3. Mediating Effect Test

Subsequently, to reveal the direct and indirect effects of employment of older people on mental health through the three aspects of individual annual income, the financial support provided to children, and support received from children, we conducted the KHB mediation analysis [33]. The method is developed for binary and logit models, but this command also includes other nonlinear probability models (ordered and multinomial) and linear regression. Meanwhile, as the model specified has more than one mediator, the question arises as to which of the mediators contributes most to the mediating effect. Thus, the KHB estimates how much of the indirect effect is contributed by each mediator. It is possible to decompose the total effect into direct and indirect effects by comparing the linear model coefficients [34]. Contrary to other decomposition methods, the KHB method gives unbiased decompositions, decomposes the impacts of both discrete and continuous variables, and extends the decomposability of linear models to nonlinear probabilistic models.

## 3. Results

### 3.1. Descriptive Analyses of Variables

Table 2 shows the main statistics of the variables. As shown in Table 2, the mean value of depression scores was 18.657. Among them, the depression score of older people employed was 17.014, less than those who did not, and this difference was statistically significant at the 1% level. About 24.77% of the sample was employed in terms of old adults, indicating that the current employment status for older Chinese people was low.

### 3.2. Basic Results Analysis

Table 3 reports the estimation results after adding different control variables, in which Models (1) and (2) are the OLS regression results, and Models (3) and (4) are ordered logit results. The OLS and ordered logit regression of Models (1) and (3) do not include any control variables, and Models (2) and (4) add variables for individual characteristics, family characteristics, and social characteristics. As shown in Table 3, employment positively impacts older adults’ mental health. That is, after controlling for individual, family, and social factors, Models (2) showed that older people who were employed had decreased depression scores of the older adults by 0.875 compared with those who did not, which was significant at the 1% level. These results were in line with Hypothesis 1 and the research of other scholars [13,16,28,35,36].

Apart from employment, several sociodemographic variables also affected the mental health of older adults. Males were likely to have better mental health status than females. As expected, the mental health of older adults with higher education was better than those with lower education. Older adults with customary marriages were less depressed than the divorced or widowed. Older adults with urban Hukou had better mental health status than those in agricultural Hukou. Those who were healthier tended to have better mental health status.

### 3.3. Robustness Test: PSM Estimation Results

**Figure 1:**
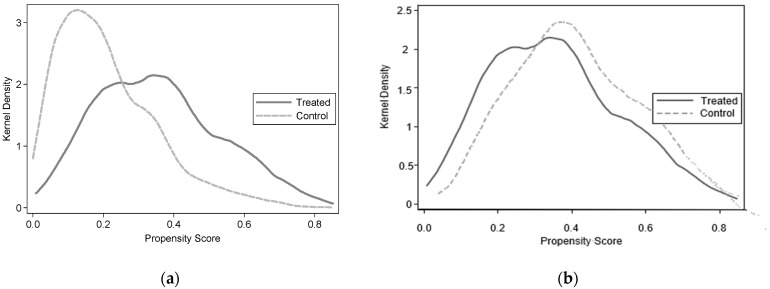
Propensity distribution of the treated and control groups before (**a**) and after (**b**) matching.

Considering there may be selection bias caused by non-random self-selection, this paper used the propensity score-matching method for robustness checks. A series of analyses were conducted to ensure the validity of the PSM method. Figure 1 shows the propensity distribution of the treated and control groups before and after matching. The results indicated that the propensity score values obtained from the model estimates before matching were significantly different between the treated and control groups. At the same time, this difference was considerably reduced after matching, implying that the overall distributions of the conditional probability to return to work were similar between the two groups.

Moreover, a balance test was conducted, and the results are shown in Table 4. Table 4 shows the results of the PSM data balanced test before and after matching. The deviation rate of all variables after matching was reduced to <10%, and the differences between all variables were no longer significant after checking, which also showed that the matching effect was great.

K-nearest neighbor matching, radius matching, kernel matching, and mahal matching methods were used to compare the treated group with the control group, and then the treatment effect was calculated. Table 5 presents the estimated results by applying the PSM method. The results in Table 5 shows that employment significantly decreased the depression score of the elderly. Namely, employment by older people was an effective way to improve mental health, which echoes the baseline result estimated by OLS and ordered logit and confirms the robustness of the results to a certain degree.

### 3.4. Effects by Sub-Groups

This study analyzed the association between employment and the mental health of older adults in different populations to inform more precise policy interventions. The sample was stratified by age, gender, education level, and household. The results are shown in Table 6.

For age, the estimation results showed that the estimated coefficient of age in the younger group (60–69 years) and (70–79 years) were −0.666 and −1.467, which were significant at the 5% level, while it was −0.882 in the older group (80 years above) and not significant. This means that employment significantly affects the mental health of the younger group but does not significantly improve the mental health status of the older group. From the perspective of gender, employment significantly increased the mental health of both male and female older people. Surprisingly, we find that the acceleration of employment on the mental health of older adults mainly affects the rural group. In terms of education level, the results showed that the positive effects of employment on the mental health of older adults mainly affected the group with a lower education background. The estimated coefficient for the lower education group were −1.019 and −0.986, significant at the 5% level, but the influence coefficient of the higher education group was not significant. Overall, the results were consistent with Hypothesis 2; that is, the impact of employment on mental health was heterogeneous in terms of individual characteristics.

### 3.5. Intermediary Mechanism Analysis

We demonstrated that employment significantly affected the mental health status of older adults. To further discuss whether older people’s employment affected their mental health through the three aspects of individual annual income, the financial support provided to children, and support received from children, the mediating effect test was conducted by using the KHB method. The results are shown in Table 7 and Table 8**.**

As shown in Table 7, Models (5)–(7) indicated that employment promoted the mental health of older adults by affecting their annual income, the financial support provided to children, and the support received from children to reduce their depression scores. The mediating mechanism showed that the relationship between mental health and employment among the elderly, individual annual income, the financial support provided to children, and support received from children could explain 81.16%, 4.95%, and 1.54% of the relationship, respectively.

The above is the estimation results, including each mediating variable. It was necessary to include all mediating variables in a model to compare the effect of mediated proportions, considering that mediating variables may be correlated with each other. As indicated in Table 8, the three mediating variables in the model explained 64.71% of employment’s effect on elders’ mental health. More specifically, the mediated proportions of individual annual income, the financial support provided to children, and support received from children were 52.97%, 7.07%, and 4.67%, respectively. In other words, employment can not only directly affect mental health for the elderly but also indirectly affect mental health through individual annual income, the financial support provided to children, and support received from children. Therefore, Hypothesis 3 was confirmed.

## 4. Discussion

Generally, the fundamental analysis and robustness test showed that employment decreased the depression of older adults and improved their mental health, supporting Hypothesis 1, which was consistent with numerous existing studies [12,14,37]. The main reasons are as follows. Firstly, the critical factor of employment of older adults is a self-financial need in China [9,22,23]. Through employment, the elderly older adults make money as financial security for their individual lives. Stable financial security is beneficial in cultivating a sense of security and reasonable expectations for the future life of the elderly, thus promoting a higher level of mental health. Secondly, older adults could extend personal relationship networks, improve information exchange, and embed themselves into the existing social system through working, thus gaining a sense of belonging [28]. Lastly, occupation is vital for achieving the individual’s internal psychological drive. Continuing to work maintains the individual’s life status and sustainable role. The individual’s self-recognition, family, and social affirmation were achieved, reducing the tendency to depression and thus improving the mental health of older adults.

Furthermore, the relationship between employment and mental health among the elderly was regulated by group differences. In the different age groups, employment significantly affects the mental health of the younger group, but it has no significant decrease in the depression of older adults. The explanation may be that 60–69-year-old seniors in better health are more likely to continue working and maintain their goals in life with better health; however, seniors above 80 years old in less good health are more concerned with intimate relationships, such as sharing the happiness of family [9,38] and pay more attention to leisure time outside of work. Therefore, promoting mental health through employment has a more pronounced effect on the younger elderly population [22]. In addition, the household registration system (Hukou) is strongly associated with depression among older adults [39]. Under the Hukou, rural older adults have limited and less access to state-funded public resources, including higher pensions, public health care, and education, and experience much more disadvantages in economic status, health status, and educational attainment than urban older adults [40]. After working, the rural elderly have a more remarkable improvement in their lives and a significant increase in their satisfaction with life, which benefits their mental health. From an educational perspective, the positive impact of employment on the mental health of older adults affects the group with lower educational backgrounds. Based on the income compensation theory proposed by Yang et al. [41], older low-education groups may enter the labor market because of financial needs. The stable income obtained from employment is conducive to reducing life pressures and promoting mental health.

Thirdly, the study suggested that individual annual income, the financial support provided to children, and support received from children played a significant mediating role in the realization of employment improving older people’s mental health. On the one hand, we showed that a career significantly boosted older adults’ annual income, which was positively related to older adults’ mental health. Generally, higher-income earners generate more positive emotions that are more conducive to mental health, which agrees with the findings of mainstream studies [9,28]. On the other hand, parents’ employment can significantly increase intergenerational support, involving the financial support provided to children and support received from children, which strongly promotes the mental health of older adults. The explanation is that employment has become an essential source of increased income for older adults, parents have continued to be necessary providers of economic resources for the family, and parents continue to provide financial support for their children as they reach adulthood to the best of their ability. Meanwhile, the reciprocal intergenerational relationship motivates children to actively provide material security, emotional interaction, and spiritual consolation to the elderly within their means. As a result, seniors’ employment improves their quality of life and reduces the financial burden on their children.

Notably, the mediated proportions of individual annual income, the financial support provided to children, and support received from children were 52.97%, 7.07%, and 4.67%, respectively. The results suggested that the mechanism of self-economic needs plays a greater role in enhancing the mental health of the elderly than the mechanism of family needs. The possible reason for this is that older people are transforming their traditional values under the continuous influences of modernity, paying more and more attention to their own subjective experiences. The logic of intergenerational reciprocity is changing from emotional to rational. Therefore, when the elderly are employed, they do not just contribute to their offspring but also consider their economic needs and self-actualization. Financial support and a sense of achievement from work are important ways to promote the mental health of the elderly. In the meantime, we must recognize another discovery in the research: although the proportion of intergenerational exchange mediated by family factors is low, the mediation still holds. Therefore, employment can, in turn, improve the mental health of older adults through intergenerational support. This indicates that family and children are still the core elements of emotional attachment and belonging for older adults, described as a “family orientation and filial piety culture” [42]. In conclusion, the proportion of mediated channels demonstrates that older adults are employed, to a great extent, for more self-economic security, yet the traditional family-centered culture still exists.

## 5. Conclusions and Policy Suggestions

### 5.1. Conclusions

Using the database of CHARLS 2018, this study shows that employment contributes to the mental health of older adults, which is more pronounced in older people who are of younger age, have a low education level, and are in rural areas. The impact of employment on the mental health of the elderly is mainly achieved by increasing individual annual income, providing financial support to children, and receiving support from children. Our research has important practical implications for actively promoting the "Healthy China Initiative" strategy, addressing population aging, and adhering to the concept of healthy aging.

### 5.2. Policy Suggestions

The government should give appropriate guidance to transform the role of the elderly as care receivers to that of producers, improve their social welfare, and safeguard their interests. First, considering the beneficial effect of employment on the mental health of the elderly, the delayed retirement policy is expected to promote the mental health of the elderly. The relevant departments should play an advocating and supporting role to encourage the employment of the elderly, such as providing skills training and entrepreneurial subsidies. Second, due to the group differences in this effect, we should focus on the employment of these groups of people, such as the elderly of younger age, low education level, and rural areas, and actively improve mental health by promoting their employment. Third, the state should formulate a policy to protect the rights of the elderly, fully implement a universal insurance plan, expand pension insurance coverage, and gradually increase essential pension insurance benefits for urban and rural residents.

### 5.3. Limitations and Prospects

Several limitations of this study will need to be further explored in future research: First, although PSM can reduce selection bias caused by self-selection to some extent, it is expected that the longitudinal design used in the future will explore the causal relationship between employment and the mental health of older adults; Second, in addition to economic and family intergenerational relationships, older adults’ attitudes toward aging, perceptions of aging, and social values may also moderate the relationship between employment and the mental health of older adults. However, the need for corresponding data makes it difficult to conduct further research, and our future research is expected to work on this.

## Figures and Tables

**Table 1:** Variables and definitions.

Variables	Definitions
Depression	Depression was measured by the depression score obtained by the CES-D scale in CHARLS 2018, with a value ranging from 10–40. The higher the score, the more pronounced the depressive symptoms.
Employment	Nonfarm employment or agricultural employment = 1, not working = 0
Age	Age.
Agen	Age squared.
Gender	Male = 1, female = 0.
Marital status	Married = 1, other = 0.
Education	Years of education: illiterate = 0; primary school = 6; junior high school = 9; senior high school = 12; junior college = 15; undergraduate = 16; graduate = 18.
Self-rated health	Self-rated health status. The higher the value, the worse the self-rated health status.
Child	The number of children.
Household annual income	The sum of household income in the past year and taking the logarithm.
Pension	Receive, expect to receive, or contribute to the pension = 1, and not having pension = 0.
Hukou	Non-agricultural household = 1, and agricultural Hukou = 0.
Individual annual income	The sum of personal income in the past year and taking the logarithm.
Financial support provided to children	The sum of financial support provided to children in the past year and taking the logarithm.
Support received from children	The number of supports provided by children in the past year, which includes emotional support, financial support, and caregiving, with a value ranging from 0–3.

**Table 2:** Descriptive statistics of the variables.

Variables	Total (*N* = 4316)		Employ = 1 (*N* = 1069)		Employ = 0 (*N* = 3247)		*t*-Test
	Mean	SD	Mean	SD	Mean	SD	
Depression	18.657	6.535	17.014	5.787	19.198	6.676	2.184 ***
Employment	0.248	0.432	1	0	0	0	-
Age	66.486	5.127	64.471	3.797	67.149	5.332	2.677 ***
Agen	44.466	7.112	41.710	5.077	45.374	7.445	3.664 ***
Gender	0.572	0.495	0.697	0.460	0.531	0.499	−0.166 ***
Marital status	0.870	0.336	0.886	0.318	0.865	0.341	−0.020 *
Education	4.627	3.876	5.535	3.971	4.328	3.798	−1.207 ***
Self-rated health	2.936	0.992	2.618	0.983	3.041	0.972	0.423 ***
Child	2.849	1.288	2.494	1.143	2.966	1.312	0.473 ***
Household annual income	7.943	3.045	7.193	3.689	8.190	2.757	0.997 ***
Pension	0.918	0.274	0.933	0.251	0.913	0.282	−0.019 **
Hukou	0.121	0.326	0.134	0.341	0.117	0.322	−0.017

Note: figures in parentheses are robust standard errors. *** *p* < 0.001, ** *p* < 0.05, and * *p* < 0.1.

**Table 3:** Results of OLS and ordered logit.

Variables	OLS				Ordered Logit			
	(1)		(2)		(3)		(4)	
	Coefficient	S.E.	Coefficient	S.E.	Coefficient	S.E.	Coefficient	S.E.
Employed	−2.184 ***	0.212	−0.875 ***	0.214	−0.578 ***	0.060	−0.239 **	0.065
Age			−0.268	0.244			−0.093	0.068
Agen			0.151	0.173			−0.056	0.048
Gender			−1.649 ***	0.209			−0.433 ***	0.061
Marital status			−1.075 ***	0.301			−0.283 ***	0.087
Education			−0.148 **	0.027			−0.045 ***	0.008
Self-rated health			2.109 ***	0.095			0.624 ***	0.029
Child			0.116	0.080			0.044 *	0.024
Household annual income			0.037	0.028			0.006	0.009
Pension			0.011	0.342			−0.012	0.100
Hukou			−0.953 ***	0.281			−0.351 **	0.087
Constant	19.198 ***	0.117	25.812 **	8.557				
*N*	4316		4316		4316		4316	
R^2^	0.021		0.173					

Note: OLS is ordinary least squares estimation. S.E. is the robust standard error, *** *p* < 0.001, ** *p* < 0.05, and * *p* < 0.1.

**Table 4:** Results of PSM data-balanced test.

Variable	Sample	Mean Value			Percentage of Reduction (%)|Bias|	*t*-Test	
		Treated Group	Control Group	% Bias		t	*p* > t
Age	Before matching	64.471	67.149	−57.8		−15.20	0.000
	After matching	64.471	64.411	1.3	97.7	0.36	0.719
Agen	Before matching	41.710	45.374	−57.5		−14.98	0.000
	After matching	41.710	41.644	1.0	98.2	0.29	0.770
Gender	Before matching	0.697	0.531	34.6		9.61	0.000
	After matching	0.697	0.693	0.7	97.9	0.18	0.860
Marital status	Before matching	0.886	0.865	6.2		1.73	0.084
	After matching	0.886	0.886	−0.2	97.0	−0.04	0.965
Education	Before matching	5.535	4.328	31.1		8.91	0.000
	After matching	5.535	5.590	−1.4	95.4	−0.32	0.748
Self-rated health	Before matching	2.618	3.041	−43.2		−12.29	0.000
	After matching	2.618	2.619	−0.0	99.9	−0.01	0.996
Child	Before matching	2.494	2.966	−38.4		−10.54	0.000
	After matching	2.494	2.530	−2.9	92.3	−0.75	0.454
Household annual income	Before matching	7.193	8.190	−30.6		−9.38	0.000
	After matching	7.193	7.196	−0.1	99.7	−0.02	0.987
Pension	Before matching	0.933	0.913	7.3		2.02	0.044
	After matching	0.933	0.934	−0.7	90.8	−0.17	0.868
Hukou	Before matching	0.134	0.117	5.1		1.45	0.146
	After matching	0.134	0.121	4.0	21.2	0.92	0.360
Sample	Ps R^2^	LR chi2	*p* > chi2	MeanBias	MedBias	B	R
Unmatched	0.128	619.540	0.000	31.2	32.8	90.8 *	0.85
Matched	0.001	4.180	0.939	1.2	0.9	8.8	0.98

Note: * if B > 25%, R outside [0.5; 2]; Ps R^2^ represents the pseudo R-squared, LR chi2 denotes the chi-square statistic, B represents the absolute standard deviation, and R denotes the standard deviation ratio.

**Table 5:** Average treatment effect of employment on the mental health of older people.

Method	Treated Group	Control Group	ATT	Standard Error
K-nearest neighbor matching	17.014	17.897	−0.883 ***	0.270
Radius matching	17.022	17.911	−0.889 ***	0.248
Kernel matching	17.014	17.916	−0.0902 ***	0.248
Mahal matching	17.014	17.845	−0.831 ***	0.226

Note: *** *p* < 0.001, and k = 4; ATT represents the average treatment effects on treated.

**Table 6:** Heterogeneity test results.

	Age							
	60 ≤ Age ≤ 69		70 ≤ Age ≤ 79		Age ≥ 80			
	Coefficient	S.E.	Coefficient	S.E.	Coefficient		S.E.	
Employed	−0.666 **	0.235	−1.467 **	0.512	−0.882		1.181	
Control variables	Yes		Yes		Yes			
*N*	3251		985		80			
R^2^	0.178		0.171		0.132			
	**Gender**				**Hukou**			
	**Female**		**Male**		**Agricultural** **household**		**Non-agricultural household**	
	**Coefficient**	**S.E.**	**Coefficient**	**S.E.**	**Coefficient**	**S.E.**	**Coefficient**	**S.E.**
Employed	−0.810 **	0.401	−0.973 ***	0.249	−0.927 ***	0.228	0.383	0.624
Control variables	Yes		Yes		Yes		Yes	
*N*	1847		2469		3793		523	
R^2^	0.152		0.128		0.163		0.204	
	**Education level**							
	**Illiterate**		**Primary school**		**Junior high school**		**Senior high school** **and above**	
	**Coefficient**	**S.E.**	**Coefficient**	**S.E.**	**Coefficient**	**S.E.**	**Coefficient**	**S.E.**
Employed	−1.019 **	0.491	−0.986 **	0.312	−0.580	0.444	−0.838	0.636
Control variables	Yes		Yes		Yes		Yes	
*N*	1226		2021		723		346	
R^2^	0.137		0.152		0.176		0.215	

Note: S.E. is the robust standard error, *** *p* < 0.001, and ** *p* < 0.05.

**Table 7:** Mediating Effects using the KHB-Method.

	(5) Individual Annual Income		(6) Financial Support Provided to Children		(7) Support Received from Children	
	Coefficient	S.E.	Coefficient	S.E.	Coefficient	S.E.
Total effect	−0.883 ***	0.222	−1.154 ***	0.305	−0.934 ***	0.232
Direct effect	−0.166	0.254	−1.096 ***	0.306	−0.920 ***	0.232
Indirect effect	−0.716 ***	0.118	−0.057 *	0.030	−0.014	0.013
Mediating effect (%)	81.16		4.95		1.54	
Control variables	Yes		Yes		Yes	
*N*	3856		2446		3601	
R^2^	0.18		0.19		0.18	

Note: S.E. is the robust standard error, *** *p* < 0.001, and * *p* < 0.1.

**Table 8:** Components of Indirect Effects using the KHB-Method.

Mediating Variables	Total Effect		Direct Effect		Indirect Effect		Mediating Effect (%)	
	Coefficient	S.E.	Coefficient	S.E.	Coefficient	S.E.	Percentage Total	Percentage Indirect
Total	−1.093 ***	0.312	−0.386	0.348	−0.707 ***	0.154	64.71	100
Individual annual income					−0.579	0.148 ^1^	52.97	81.85
Financial support provided to children					−0.077	0.048 ^1^	7.07	10.93
Support received from children					−0.051	0.031 ^1^	4.67	7.22

Note: S.E. is the robust standard error, *** *p* < 0.001. ^1^ S.E. are standard errors.

## Data Availability

We obtained the CHARLS data from the website http://charls.pku.edu.cn (accessed on 6 October 2022), which is available to users worldwide.
